# Differences of liver CT perfusion of blunt trauma treated with therapeutic embolization and observation management

**DOI:** 10.1038/s41598-020-76618-w

**Published:** 2020-11-12

**Authors:** Yon-Cheong Wong, Li-Jen Wang, Cheng-Hsien Wu, Huan-Wu Chen, Kuo-Ching Yuan, Yu-Pao Hsu, Being-Chuan Lin, Shih-Ching Kang

**Affiliations:** 1 Emergency and Critical Care Radiology, Department of Medical Imaging and Intervention, Chang Gung Memorial Hospital, Chang Gung University, 5 Fu-Hsin Street, Gueishan, Taoyuan, 333 Taiwan; 2 Division of Trauma and Emergency Surgery, Department of Surgery, Chang Gung Memorial Hospital, Chang Gung University, Taoyuan, Taiwan

**Keywords:** Diseases, Gastroenterology

## Abstract

Massive hepatic necrosis after therapeutic embolization has been reported. We employed a 320-detector CT scanner to compare liver perfusion differences between blunt liver trauma patients treated with embolization and observation. This prospective study with informed consent was approved by institution review board. From January 2013 to December 2016, we enrolled 16 major liver trauma patients (6 women, 10 men; mean age 34.9 ± 12.8 years) who fulfilled inclusion criteria. Liver CT perfusion parameters were calculated by a two-input maximum slope model. Of 16 patients, 9 received embolization and 7 received observation. Among 9 patients of embolization group, their arterial perfusion (78.1 ± 69.3 versus 163.1 ± 134.3 mL/min/100 mL, *p* = 0.011) and portal venous perfusion (74.4 ± 53.0 versus 160.9 ± 140.8 mL/min/100 mL, *p* = 0.008) were significantly lower at traumatic parenchyma than at non-traumatic parenchyma. Among 7 patients of observation group, only portal venous perfusion was significantly lower at traumatic parenchyma than non-traumatic parenchyma (132.1 ± 127.1 vs. 231.1 ± 174.4 mL/min/100 mL, *p* = 0.018). The perfusion index between groups did not differ. None had massive hepatic necrosis. They were not different in age, injury severity score and injury grades. Therefore, reduction of both arterial and portal venous perfusion can occur when therapeutic embolization was performed in preexisting major liver trauma, but hepatic perfusion index may not be compromised.

## Introduction

Liver is one of the most frequently injured abdominal organs following blunt abdominal trauma. Immediate mortality is caused by uncontrolled liver hemorrhage and therefore it is best treated with damaged control surgery^1,2^. However, if the patients are hemodynamically stable and do not have concurrent bowel perforation, they are usually treated with non-operative management (NOM)^3,4^. Failures of NOM are usually caused by continuous silent liver hemorrhage^5^. It has been reported that regardless of the grades of liver trauma, continuous silent liver hemorrhage can often be treated by adjunct embolization^3,5–7^. The success rates of non-operative management are generally greater than 90% when embolization is incorporated into the management algorithm as a means to salvage NOM failures^3,7^.

Potential delayed complications of embolization for liver trauma are massive liver necrosis, liver abscess and biloma^8–13^. Among them, massive hepatic necrosis is a major concern among trauma surgeons because the death of a large number of contiguous hepatocytes can contribute to a high morbidity and late mortality^8,9^. Moreover, as contradictory to what is otherwise a planned NOM, patients of massive hepatic necrosis usually require multiple operations and long hospital stay. The reported complication rate of massive hepatic necrosis, however, is contentious and varies in different publications^8,9,13^.

Liver has a unique dual arterial and portal venous blood supply that provides protection against devascularized ischemia. Our hypothesis is that therapeutic embolization of the liver alone does not cause devascularization injury. We presume that liver devascularization injury can occur only when portal venous system and hepatic arterial system are concurrently disrupted. Complications of liver devascularization injury can vary from massive hepatic necrosis to a spectrum of liver perfusion defect. These changes in devascularization injury cannot be quantified on a regular contrast-enhanced CT^14–16^. With the advent of new technology, liver CT perfusion (CTP) which is a sequential volumetric data of the liver can be obtained readily using a rapid volumetric CT scanner^17–19^. This is a method to trace the temporal changes in CT enhancement of volume tissue of interest after intravenous administration of iodinated contrast medium^17–21^. To date, there is no related investigation of liver CTP on liver trauma and embolization yet. Therefore, we perform CTP on major liver trauma to clarify whether or not our hypothesis regarding therapeutic liver embolization alone dose not cause liver devascularization injury is true.

## Results

**Figure 1:**
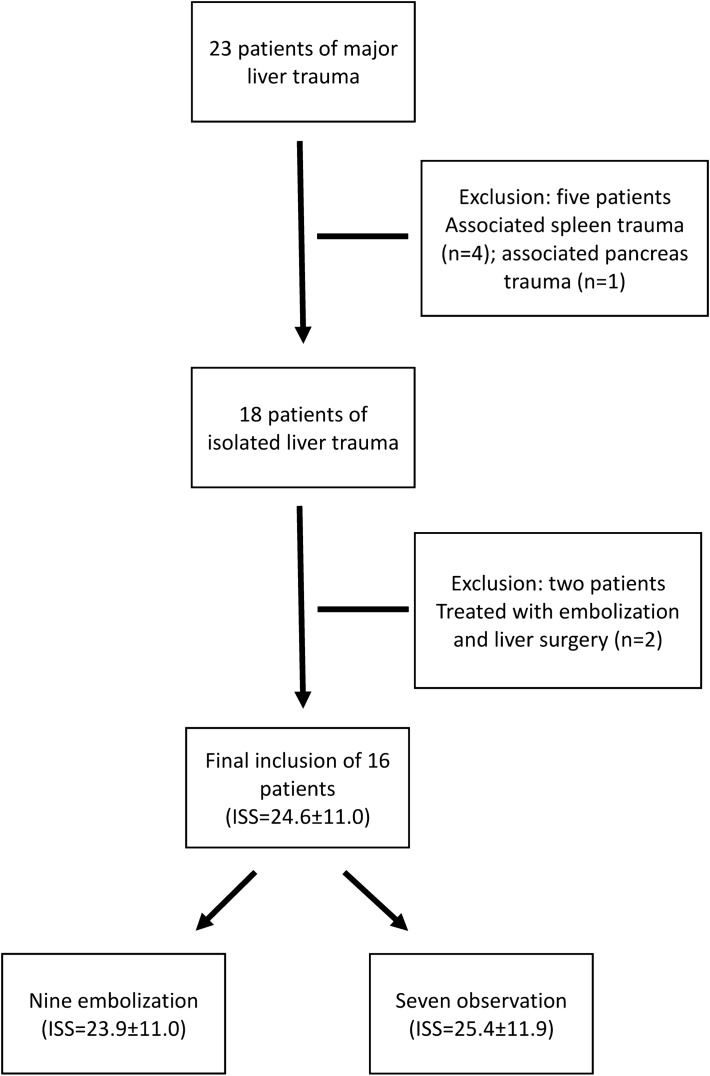
Flowchart of patient inclusion. After exclusion of five patients with other associated abdominal organs injuries and two patients with liver surgery in addition to embolization, the final inclusion was sixteen patients. *ISS* injury severity score.

A total of 23 patients of major liver trauma who were assigned in NOM category fulfilled the study criteria underwent liver CTP examinations (Fig. 1). Five were excluded because they were associated with other major abdominal organs trauma such as spleen (n = 4) and pancreas (n = 1). Two were excluded because of NOM failure and subsequently treated with adjunct liver surgery. The final inclusion comprised 16 patients (6 women, 10 men) with a mean age of 34.9 ± 12.8 years. The mean injury severity score of the 16 patients was 24.6 ± 11.0. In accordance with the American Association for the Surgery of Trauma, there were six grade III liver trauma and ten grade IV liver trauma patients.

Of the 16 patients, 7 received observation management, 9 received embolization management. Eight embolization therapies were performed at right hepatic artery. One embolization therapy was performed at both right hepatic artery and middle hepatic artery. Gelfoam pledget was the main embolic agent used in the embolization. Among them, metallic microcoils were added to five patients for a better hemostasis at the discretion of operators. Between patients receiving observation and embolization, differences of the mean age (32.3 ± 13.3 years versus 36.9 ± 12.8 years), mean injury severity score (25.4 ± 11.9 versus 23.9 ± 11.0) and mean liver injury grades (3.43 ± 0.54 versus 3.78 ± 0.44) did not differ significantly. Vital signs and blood tests on arrival at emergency department between the two groups also did not differ (Table 1).

**Table 1:** Vital signs and blood tests between major liver trauma patients receiving observation management and intervention management.

Items		Observation (n = 7)	Intervention (n = 9)	*p* value*
Heart rate	bpm	100.6 ± 24.2	97.0 ± 15.1	0.672
Systolic blood pressure	mmHg	130.0 ± 22.7	115.0 ± 16.5	0.204
Revised trauma score		7.5 ± 0.8	7.6 ± 0.5	0.816
pH		7.371 ± 0.085	7.363 ± 0.070	0.477
Hemoglobin	g/dL	11.9 ± 1.7	11.8 ± 2.3	1.000
Platelet count	× 10^3^/µL	236.0 ± 53.7	198.7 ± 81.4	0.071
INR		1.17 ± 0.15	1.23 ± 0.24	0.786
ER blood transfusion				0.596^#^
Present	Patient (%)	4 (57.1%)	7 (77.8%)	
Absent	Patient (%)	3 (42.9%)	2 (21.2%)	

*All *p* values of continuous data are calculated using Mann–Whitney test except a categorical data (#) is calculated using Fisher’s exact test.

*bpm* beats per minute, *INR* international normalized ratio, *ER* emergency room.

**Figure 2:**
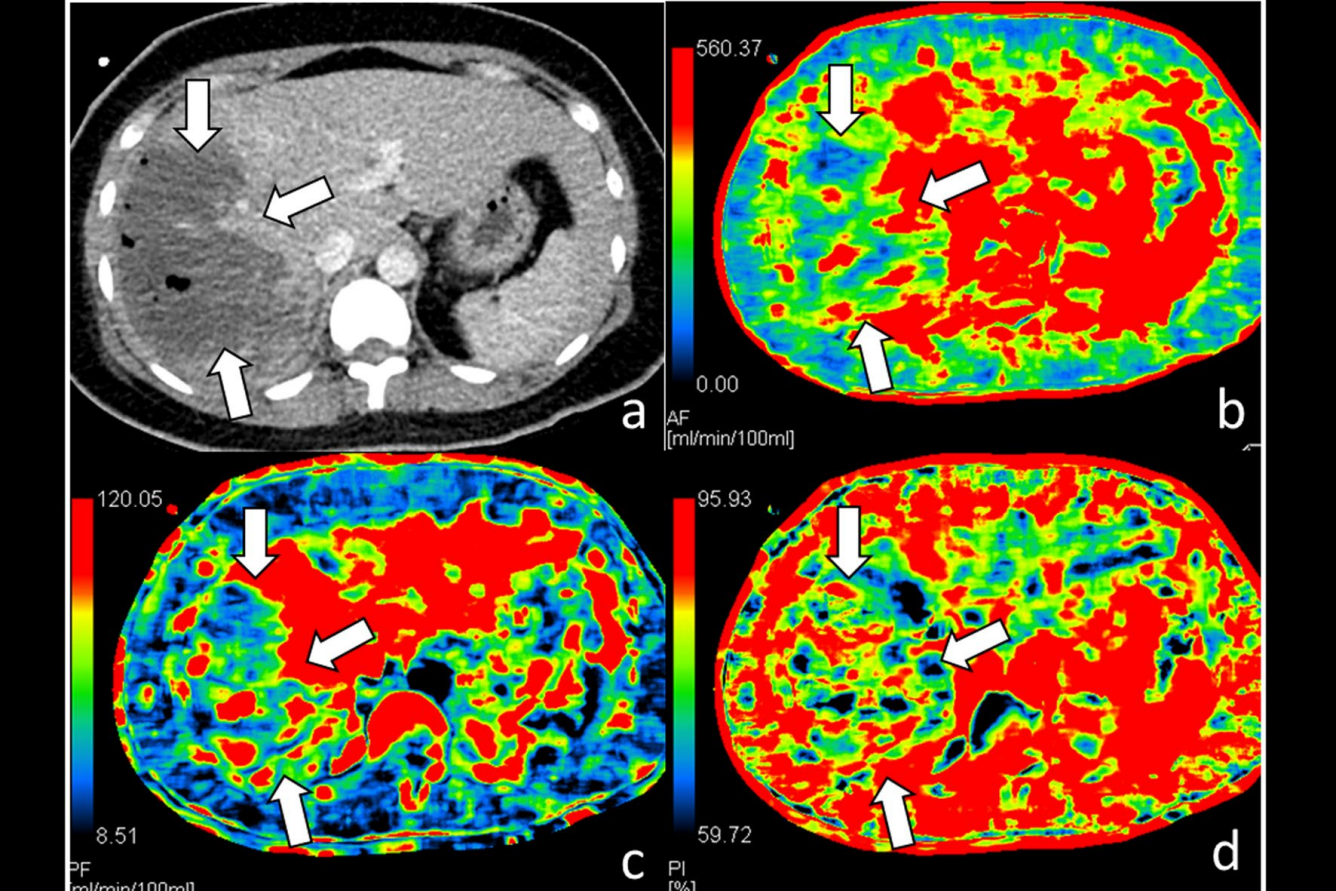
CT of a 34-year-old woman with grade IV liver injury at right lobe after embolization management. (**a**) Contrast-enhanced CT of the liver shows extensive right lobe injury (arrows); (**b**) hepatic arterial perfusion CT and (**c**) hepatic portal venous perfusion CT show decreased arterial and portal venous flows to traumatic parenchyma at right lobe (arrows); (**d**) hepatic perfusion index which is a ratio of arterial perfusion to the total hepatic perfusion is normal at traumatic parenchyma (arrows).

Liver CTP examinations of all patients were performed on a mean of 9.6 ± 3.6 days after blunt liver trauma and successfully treated with NOM. The mean radiation dose of liver CTP was 12.0 ± 2.5 millisievert. Among 9 patients of embolization group, their whole liver hepatic arterial perfusion (HAP) 78.1 ± 69.3 versus 163.1 ± 134.3 mL/min/100 mL, *p* = 0.011, and portal venous perfusion (PVP) 74.4 ± 53.0 versus 160.9 ± 140.8 mL/min/100 mL, *p* = 0.008 were significantly lower at traumatic parenchyma than at non-traumatic parenchyma (Table 2). However, the difference of whole liver hepatic perfusion index (HPI) between trauma and non-traumatic parenchyma did not differ (Fig. 2).

**Table 2:** Paired comparison of whole liver CTP parameters between traumatic liver parenchyma and non-traumatic liver parenchyma among patients receiving intervention management.

Items		Trauma (n = 9)	Non-trauma (n = 9)	*p* value*
HAP	mL/min/100 mL	78.1 ± 69.3	163.1 ± 134.3	0.011
PVP	mL/min/100 mL	74.4 ± 53.0	160.9 ± 140.8	0.008
HPI	HAP/(HAP + PVP)%	53.4 ± 23.9	50.6 ± 24.5	0.173

*Wilcoxon signed ranks test.

*CTP* CT perfusion, *HAP* hepatic arterial perfusion, PVP portal venous perfusion, *HPI* hepatic perfusion index.

**Figure 3:**
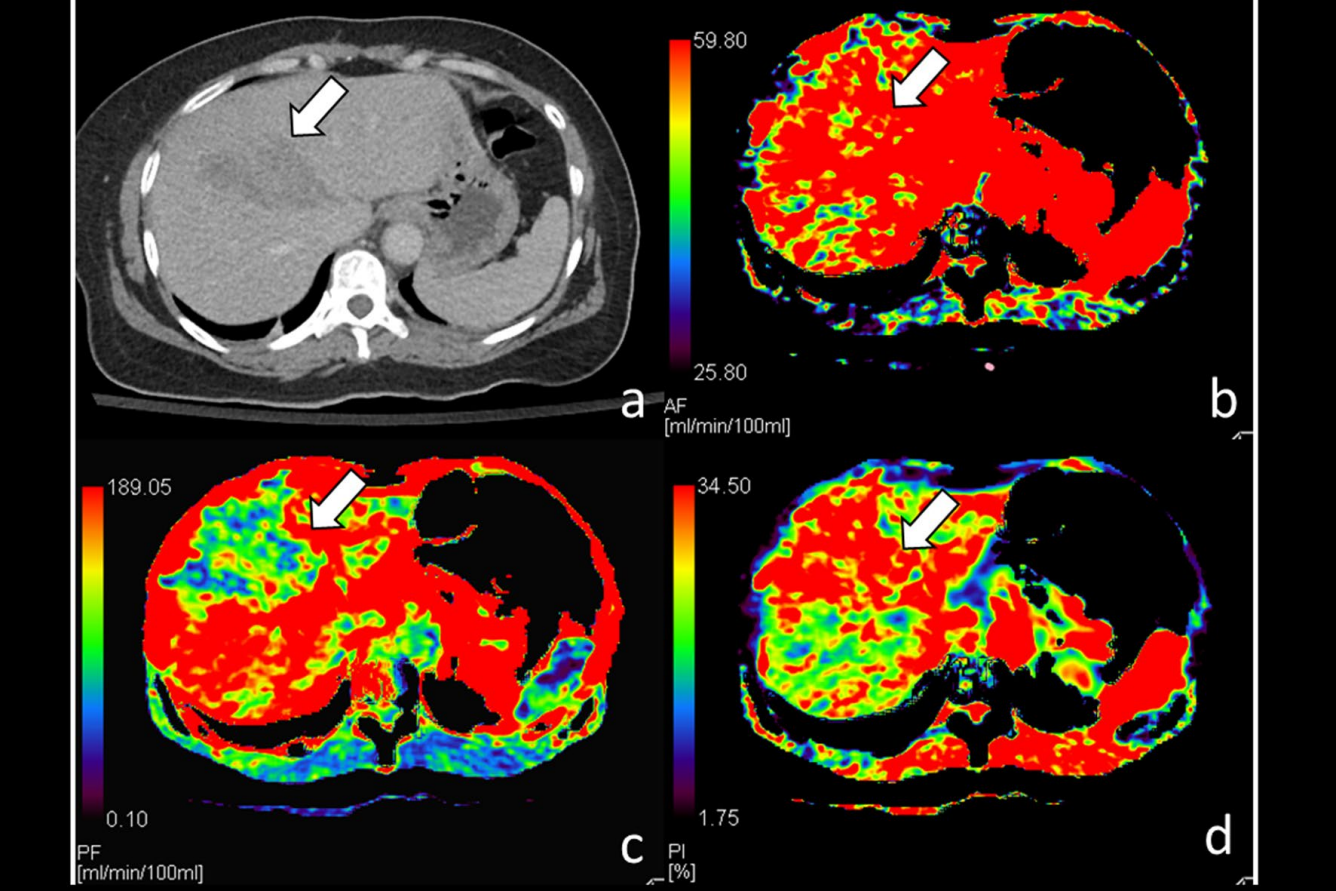
CT of a 56-year-old woman with grade III liver injury at segment IV receiving observation management only. (**a**) Contrast-enhanced CT of the liver shows focal injury at segment IV (arrow); (**b**) hepatic arterial perfusion CT does not show arterial flow defect (arrow) but (**c**) hepatic portal venous perfusion CT reveals decreased portal venous flow at traumatic parenchyma (arrow); (**d**) CT perfusion shows compensatory increase of hepatic perfusion index at segment IV (arrow).

In contrast, among 7 patients who received observation management, only whole liver PVP (132.1 ± 127.1 versus 231.1 ± 174.4 mL/min/100 mL, *p* = 0.018) was significantly lower at traumatic liver parenchyma than non-traumatic liver parenchyma (Table 3). Moreover, differences of both HAP and HPI at traumatic parenchyma and non-traumatic parenchyma were not significant among patients in this observation management group (Fig. 3).

**Table 3:** Paired comparison of whole liver CTP parameters between traumatic liver parenchyma and non-traumatic liver parenchyma among patients receiving observation management.

Items		Trauma (n = 7)	Non-trauma (n = 7)	*p* value*
HAP	mL/min/100 mL	91.9 ± 59.4	100.9 ± 73.7	0.612
PVP	mL/min/100 mL	132.1 ± 127.1	231.1 ± 174.4	0.018
HPI	HAP/(HAP + PVP)%	47.8 ± 23.8	37.1 ± 24.1	0.310

*Wilcoxon signed ranks test.

*CTP* CT perfusion, *HAP* hepatic arterial perfusion, *PVP* portal venous perfusion, *HPI* hepatic perfusion index.

For the non-traumatic liver parenchyma region, differences of HAP, PVP and HPI between embolization management group and observation management group were not statistically significant (Table 4).

**Table 4:** Comparison of whole liver CT perfusion parameters between observation management and intervention management groups at non-traumatic liver parenchyma.

Liver CTP		Observation (n = 7)	Intervention (n = 9)	*p* value*
HAP	mL/min/100 mL	100.9 ± 73.7	163.1 ± 134.3	0.266
PVP	mL/min/100 mL	231.1 ± 174.4	160.9 ± 140.8	0.368
HPI	HAP/(HAP + PVP)%	37.1 ± 24.1	50.6 ± 24.5	0.315

*Mann–Whitney test.

*CTP* CT perfusion, *HAP* hepatic arterial perfusion, *PVP* portal venous perfusion, *HPI* hepatic perfusion index.

There was no mortality is this study. Among 16 patients, none developed massive liver necrosis. Two patients developed infected bilomas and were successfully drained by percutaneous catheter. Development of biloma was not significantly associated with embolization or observation. It was also not significantly associated with liver CTP.

## Discussion

Complications of blunt liver trauma such as hepatic necrosis, liver abscess and biloma have been reported previously^8–13^. Among them, the most concerning complication is hepatic necrosis. The rate of hepatic necrosis has been reported to range from 0 to 42% after blunt liver trauma^8,9,13^. According to a study by Dabb et al., patients with hepatic necrosis had higher grade of liver injuries and were more likely to have undergone damage control surgery in addition to embolization^8^. However, these complications may occur regardless of whether blunt liver trauma is treated with observational, embolization or surgery^8–13^. In this study, only two patients developed infected biloma and was successfully treated with percutaneous catheter drainage.

Theoretically, massive hepatic necrosis is the death of a large number of hepatocytes and is related to cellular ischemia^8,9,22,23^. However, liver has a unique dual blood supply and therefore can provide protection against devascularized ischemia. Occurrence of massive hepatic necrosis must have been caused by disruption at the same time of both the arterial and portal venous systems. Patients who have massive liver necrosis usually present with severe elevation of serum liver enzymes activities and bilirubin^22,23^. In severe cases, patients can even suffer from liver organ failure^22,23^. Our patients were severely injured with a mean injury severity score of 24.6 ± 10.8 and a mean liver injury grade of 3.63 ± 0.50. None of the patients in this study developed significant hepatic necrosis nor persistent elevation of their liver enzymes for more than one week. On the contrary, their liver enzymes quickly subsided within one week. Consequently, less severe hepatic necrosis can be underestimated if the diagnosis is based only on prolonged elevation of serum liver enzymes and bilirubin.

Patients of liver trauma are usually followed up by either sonography or regular contrast-enhanced CT, under which only the organs anatomy is evaluated^14–16^. These examinations fall short of assessing the perfusion function of the injured organs. In this study, we advocate the utilization of liver CTP to detect perfusion function changes of traumatic liver parenchyma. In the past, dynamic multiple-phase contrast-enhanced CT can give a measurement of CT number changes in a region of interest thus providing a way to calculate blood flow and organ perfusion^17–21^. However, the low acquisition speed of old CT scanners and the high effective radiation dose of a serial CT scanning to the patient were the major concerns and therefore have dissuaded radiologists to use it for clinical purpose. With the advent of 320-detector CT, we can scan the upper abdomen with a maximum width of 16 cm in the z-direction in a single rotational acquisition. We can dramatically reduce the effective radiation dose of CTP without compromising image quality^24,25^ by utilizing a tube voltage of 80 or 100 kVp along with a tube current of 50–120 mAs. In addition, the noise artifact resulted from a low radiation technique is readily removed by a robust iterative reconstruction algorithm^24,25^. In our study, the mean radiation dose of liver CTP can be as low as 12.0 ± 2.5 mSv. Therefore, a 16 cm volumetric CTP by means of serial rotational acquisitions at the liver during administration of iodinated contrast medium can be obtained with a little additional amount of effective radiation dose. The quantitative maps of tissue perfusion can then be created from cine CT data and displayed on a color map scale^17–19^.

Among patients of major liver trauma treated with observation management, only PVP was significantly lower at traumatic liver parenchyma than non-traumatic liver parenchyma. These findings are consistent with a disrupted portal venous flow in the event of trauma. In contrast, among patients who received embolization management, not only PVP was lower, the HAP was also lower at traumatic liver parenchyma than those at non-traumatic liver parenchyma. These results confirmed that arterial perfusion to the traumatic liver parenchyma was further compromised if the arterial supply was blocked in the events of therapeutic embolization when portal venous flow was already disrupted.

Non-hyperselective liver embolization does not only aim at traumatic parenchyma alone, it also causes arterial occlusion to non-traumatic parenchyma which is supplied by the same artery. If arterial flow alone is blocked by embolization while the portal venous flow is intact, then liver perfusion may not be compromised. Our data have proven that at the non-traumatic liver parenchyma, neither embolization nor observation management had resulted in significant differences in HAP or PVP parameters.

Fortunately, none of our cases developed massive hepatic necrosis. It was reflected on HPI which is a relative contribution of the arterial inflow versus total vascular inflow expressed as a ratio of arterial perfusion to the total hepatic perfusion. In this study, HPI of traumatic liver parenchyma was slightly higher than that of non-traumatic liver parenchyma in various group comparisons, even though the differences did not reach statistical significance. In other words, the slight increase of HPI at traumatic parenchyma represented a general perfusion compensation to the liver after a successful hemostasis. This compensatory microcirculation phenomenon has also been reported in a porcine poly-trauma model with hemorrhagic shock^26^.

We acknowledge that one of the limitations of this study was a small sample size. Even though the number was small, this study had already confirmed our hypothesis that therapeutic embolization of the liver alone does not cause devascularization injury. Our subjects were enrolled over a long period of time was our second limitation. However, this did not affect our results. Placement of region of interests (ROIs) at each hepatic segment and traumatic parenchyma was important for measurements of CTP parameters. Therefore, for the purpose of ensuring our technologists of placing ROIs correctly, a radiologist investigator oversaw the placement of ROIs in every case.

## Conclusion

At the time of major liver trauma, portal vein was disrupted as a result of parenchymal lacerations. Therapeutic liver embolization for traumatic hemorrhage by occluding hepatic arteries reduced arterial flow to the supplying territory. However, only when both events occurred concurrently, then HAP and PVP to traumatic liver parenchyma were compromised. Nonetheless, HPI which was a ratio of the arterial inflow versus total vascular inflow was not affected presumably rescued by compensatory microcirculation. A larger scale of investigation is needed to support this phenomenon in traumatic liver hemorrhage.

## Materials and methods

### Subjects

This prospective study was approved by Chang Gung Memorial Hospital’s institution review board (RE: 103-5849C) and all methods were carried out in accordance with relevant guidelines and regulations. Written informed consent was obtained from every participant. From January 2013 to December 2016, blunt liver trauma patients who met the following criteria were enrolled in this study. The inclusion criteria were (1) age ≥ 20 years, (2) major blunt liver trauma (grade III and IV), (3) NOM patients who had been treated by observation or embolization, (4) stable for transportation to CT examination room, (5) conscious clear and tolerable to breath-hold CT scanning (6) fully understood the study and provided written informed consents. The exclusion criteria were (1) allergy to iodinated contrast medium, (2) expected glomerular filtration rate < 45 mL/min/1.73 m^2^, (3) no satisfactory antecubital venous access for an 18 gauge cannula, (4) pregnancy.

### Recording of demographics, liver injury grades, embolization techniques, vital signs and blood tests data

Demographic data of the included patients including sex, age, injury severity score were recorded. Severity of liver trauma on CT examinations was graded in accordance with the scales of American Association for the Surgery of Trauma^27^. Our center had been practicing selective embolization for liver trauma in general and performed superselective embolization only for traumatic A-P shunts. Reviews of the embolization techniques and embolic agents were done on angiography and radiology reports. Embolization techniques were classified into selective right hepatic artery embolization, middle hepatic artery embolization and left hepatic artery embolization. Whether or not gelfoam pledget alone was used or a combination of gelfoam pledget and metallic mirocoils was used as embolic agents were searched for. Charts were reviewed for vital signs (heart rate, systolic blood pressure, revised trauma score and blood transfusion at emergency room) and blood tests data (arterial blood pH, hemoglobin level, platelet count and international normalized ratio).

### Liver CTP acquisition technique

The liver CTP of all patients were performed with a 320-detector CT scanner, Aquilion ONE (Toshiba Medical Systems Corporation, Ottawara, Japan). Patients were prepared with oxygen hyperventilation through a mask at a flow of 10 L/min for five minutes before and during the examination to support a longer breath-hold sequence. Slices for CTP were selected from pre-contrast helical scans to include the whole liver. A venous catheter (18–20 gauge) was placed in the antecubital vein and 50 mL of nonionic contrast material (Omnipaque 350; GE HealthCare Ireland, Ireland) was administered at a rate of 6 mL/second with a power injector, followed by 30 mL of saline chaser.

Breath holding dynamic scans were performed 10 s after injection of contrast material started. A series of low radiation dose sequential volumetric scans were performed at every 2 s for 28 s, every 4 s for 24 s, every 8 s for 32 s. The scanning parameters were 100 kV, 90 mA, 0.5 s/rotation, 0.5 mm thickness, 320 slices, 512 × 512 matrices.

The dose length product for perfusion CT were recorded from the dose summary page generated by CT scanner. The effective radiation dose in millisievert was calculated by multiplying the total dose length product by the coefficient of 0.015 proposed by the International Commission on Radiological Protection.

### Recording of liver CTP parameters

Liver CTP parameters including the HAP, PVP and HPI were calculated on a pixel-by-pixel basis by using the two-input maximum slope model, in which the results were expressed in mL per minute per 100 mL (mL/min/100 mL). Artefacts from respiratory mis-registrations between multiple acquisitions were compensated by registration techniques software. Oval-shaped ROIs were placed on the abdominal aorta at the level of the celiac axis, main portal vein, liver and spleen to generate a time-density curve. The ROIs of the liver were sized as large as possible to minimize the effect of image noise while avoiding inclusion of macroscopically visible vessels. The ROIs were placed on each segment (S2 to S8) of the liver on every image. Segment 1 was excluded because of its small size and inconsistent measurement. The values of HAP, PVP and HPI of each segment on every image were calculated to obtain the mean segmental liver CTP parameters. The whole liver CTP parameters were the mean of the summation of segmental liver CTP parameters.

### Records of liver trauma-related complications

A monthly research meetings attended by radiologists and trauma surgeons were conducted to determine if the patients had developed liver-related complications such as massive liver necrosis or infected liver collection. The final determination on trauma-related complications of each participant was obtained based on a consensus decision. Massive liver necrosis was defined as liver dysfunction with marked elevation of serum aminotransferase and bilirubin levels for more than one week. Infected liver collection, either an abscess or infected biloma, was defined as an abnormal cystic liver collection with elevation of white blood cell count and fever. Patients who fulfilled none of the above criteria were considered free from complications.

### Data analyses and statistics

Descriptive statistics was used to present radiation dose values. We used Mann–Whitney U-test to compare liver CTP parameters between groups and Wilcoxon Signed Ranks test for paired-comparison within the same groups. A 2-tail *p* value < 0.05 was considered statistically significant.

## Data Availability

The datasets generated during and/or analysed during the current study are not publicly available due to institutional policy but are available from the corresponding author on reasonable request.
